# Drought-Conditioning of Quaking Aspen (*Populus tremuloides* Michx.) Seedlings During Nursery Production Modifies Seedling Anatomy and Physiology

**DOI:** 10.3389/fpls.2020.557894

**Published:** 2020-09-04

**Authors:** Joshua L. Sloan, Owen T. Burney, Jeremiah R. Pinto

**Affiliations:** ^1^ John T. Harrington Forestry Research Center, New Mexico State University, Mora, NM, United States; ^2^ Department of Forestry, New Mexico Highlands University, Las Vegas, NM, United States; ^3^ Rocky Mountain Research Station, Forest Service, U.S. Department of Agriculture, Moscow, ID, United States

**Keywords:** *Populus tremuloides*, drought, irrigation, drought-conditioning, *δ*^13^C, xylem

## Abstract

In the western US, quaking aspen (*Populus tremuloides* Michx.) regenerates primarily by root suckers after disturbances such as low to moderate severity fires. Planting aspen seedlings grown from seed may provide a mechanism to improve restoration success and genetic diversity on severely disturbed sites. However, few studies have examined the use of container-grown aspen seedlings for restoration purposes from both the outplanting and nursery production perspective. Thus, the purpose of this novel study was to examine how alterations in irrigation levels during nursery production across three seed sources would impact seedling performance attributes on harsh, dry outplanting sites. Irrigation treatments were based on three irrigation levels, determined gravimetrically: High = 90%, Medium = 80%, and Low = 70% of container capacity. The three seed sources represented a latitudinal gradient across the aspen range (New Mexico, Utah, and Alberta). Carbon isotope analysis indicated irrigation treatments were effective in creating higher levels of water stress for both the Low and Medium irrigation levels compared to seedlings under the High irrigation level. Seedlings subject to the Low irrigation level were found to induce greater height, higher photosynthetic rates, larger percentages of hydraulically active xylem, and faster xylem flow velocities compared to the High irrigation level. The lack of an interaction between irrigation treatments and seed source for nearly all response variables suggests that nursery conditioning *via* irrigation limitations may be effective for a range of aspen seed sources.

## Introduction

There is a growing global demand for the restoration of degraded forest ecosystems. However, many of these restoration efforts often overlook nursery systems and the associated cultural practices that are critical to the success of these restoration activities (Haase and Davis, 2017). One species of restoration interest is quaking aspen (*Populus tremuloides* Michx.), which is a widely distributed tree species in North America that provides a variety of important values, including commercial timber production, wildlife habitat, and recreational activities (Landhäusser et al., 2019). However, aspen is currently in decline throughout much of its range due to a variety of factors including large, high-severity fires and severe droughts (Rehfeldt et al., 2009). This decline is likely to accelerate as future climatic conditions are projected to be warmer and drier (Rehfeldt et al., 2009; Allen et al., 2015).

In the western US, post-fire environments and mine reclamation sites are typical targets for forest restoration activities. Currently, land managers attempting to restore aspen on these disturbed sites rely almost entirely on aspen’s ability to regenerate vegetatively *via* root suckering (Long and Mock, 2012). Reliance solely on vegetative reproduction limits genetic diversity and thus reduces resiliency and the potential for adaptations to future stresses (Ally et al., 2010). On the other hand, sexual reproduction *via* seed is rare across the range of aspen, hindering both the maintenance of genetic diversity and overall regeneration (Long and Mock, 2012; Fairweather et al., 2014). Thus, there has been a growing interest in restoring quaking aspen *via* artificial regeneration throughout its range, although this is not currently practiced in the western US (Howe et al., 2019; Landhäusser et al., 2019). Planting nursery-grown seedlings produced from collected seed will likely improve the level of genetic diversity at aspen restoration sites that would not likely occur otherwise under natural conditions, especially with a changing climate (Eriksson, 1992; Landis et al., 2003).

Most of the research connected to aspen nursery cultural practices was developed in western boreal Canada in relation to mine reclamation in the region (Macdonald et al., 2012; Howe et al., 2019). Results from some of these studies have shown that increasing both root:shoot and non-structural carbohydrates through manipulation of nursery practices improved outplanting success under a range of environmental conditions (Landhäusser et al., 2012a; Landhäusser et al., 2012b). However, a recent study found that using these same nursery cultural practices from Landhäusser et al. (2012a; 2012b) resulted in a range of morphological and phenological responses based on genetic influences from three distinct seed sources (Howe et al., 2019). A continent-wide genetic survey of aspen revealed that there are two distinct genetic clusters: a southern and northern cluster in which the southern cluster contains higher rates of triploidy occurrence among genets (Mock et al., 2012; Callahan et al., 2013). Structural and physiological differences have been observed with triploidy and polyploidy plant species including increases in cell size (*e.g.*, larger xylem elements) which may result in a higher likelihood of xylem cavitation under drought conditions (Mock et al., 2012; Greer et al., 2018). These findings suggest that continued research is necessary to improve aspen nursery protocols for a range of genetic sources that will result in improved outplanting success across genotypes.

Soil moisture is often one of the more limiting factors on post-fire and mine reclamation sites, resulting in negative effects on plant physiology and overall seedling performance (Galmés et al., 2007; Grossnickle, 2012; Claeys and Inzé, 2013; Flathers et al., 2016). Moisture stress in plants can lead to xylem cavitation, embolism, and ultimately mortality (Schreiber et al., 2015). Thus, it is critical to produce seedlings in the nursery that are capable of withstanding higher levels of water stress. This, in part, can be accomplished through nursery conditioning based on the Target Plant Concept (TPC). The TPC focuses on identifying and manipulating those seedling traits produced in the nursery that improve seedling performance for specific outplanting conditions (Landis, 2011; Dumroese et al., 2016). Water stress conditioning in the nursery as a treatment to help seedlings establish on sites with dry conditions has been done on a number of different species, often with mixed results (van den Driessche, 1991; Villar-Salvador et al., 1999; Vilagrosa et al., 2003; Villar-Salvador et al., 2013). On moisture limited planting sites, one major area of interest is the alteration of xylem structure and function so as to improve an individual seedling’s ability to buffer itself against water stress and drought-induced mortality (Venturas et al., 2017).

Water is transported through plants *via* secondary xylem elements (*i.e.*, tracheids in gymnosperms and vessels in angiosperms). Primary xylem transitions to secondary xylem shortly (*i.e.*, a few weeks) after germination depending on the species (Miller and Johnson, 2017). Based on the TPC, it is hypothetically possible to condition seedlings in the nursery to tolerate higher levels of water stress by limiting soil moisture through reductions in irrigation regimes at a time when secondary xylem is initiating for the seedling (Miller and Johnson, 2017). Exposing seedlings to stress at this time of xylem transition is hypothesized to alter the secondary xylem structure and function, influencing such characteristics as xylem element diameter and the total amount of hydraulically active xylem. For example, narrower xylem elements are more resistant to cavitation and thus more likely to tolerate higher levels of drought stress (Olson and Rosell, 2013). Additionally, building a greater buffer against drought stress may also be a function of increasing the total number of xylem elements that are hydraulically conductive (Jacobsen et al., 2015).

The main objective of this novel study was to examine the effects of drought conditioning treatments *via* irrigation limitation in the nursery across three seed sources of *Populus tremuloides*, with a focus on the effects these treatments had on seedling morphology and physiology. In order to assess the impacts of irrigation limitations on seedling development, a comprehensive suite of morphological and physiological parameters were assessed which were expected to either be directly related to drought tolerance (*e.g.*, xylem structure and function) or which are considered to be standard measures of nursery seedling quality (*e.g.*, height, root collar diameter, seedling biomass, *etc.*; see Haase, 2008). Specifically, we hypothesized that: 1) drought-conditioned seedlings would have smaller vessel diameters and greater percentages of hydraulically active xylem; 2) southern seed sources would have inherently smaller vessel diameters and a greater percentage of hydraulically active xylem; and 3) there would be no interaction between seed source and drought conditioning treatments.

## Materials and Methods

### Seedling Production and Experimental Treatments

The seed used in this study represented three sources of quaking aspen (*Populus tremuloides* Michx.). They were the same sources used by Howe et al. (2019) and represented a latitudinal gradient within the quaking aspen range (New Mexico, USA; N 36°24′, W 105°35′; Utah, USA; N 41°56’, W 111°31′; and Alberta, Canada; N 56°43′, W 113°31′). These sources represent different genetic clusters associated with aspen (Mock et al., 2012; Callahan et al., 2013). Within each source region, there is still a significant amount of genetic variation given collections were from 7 to 15 open pollinated clones that were spaced between 0.4 and 15 km apart. Mean annual temperature and precipitation for these locations are as follows: New Mexico, 8.5°C and 324 mm; Utah, 4.1°C and 1061 mm; and Alberta, 0.4°C and 448 mm (PRISM Climate Group, 2015; The Climate Atlas of Canada, 2019). On 26 June 2015, seeds were sown into 164 ml containers and placed in racks with 98 container capacities (Ray Leach Cone-tainers-SC10 Super, RL98 Tray, Stuewe & Sons, Inc., Tangent, OR, USA) Media was a 2:1:1 mixture of sphagnum peat, perlite, and vermiculite (v:v:v). Twelve racks were sown for each seed source (total of 1,176 seedlings); each rack represented an experimental treatment unit with each seedling representing a sub-sample. After sowing, racks were placed in a greenhouse where they received regular misting until germination. Racks of germinated seedlings were then watered to container capacity with clear water *via* overhead irrigation, and rack weights at container water-holding capacity were recorded. Prior to the initiation of irrigation treatments (described below), racks were watered when their weights dried to 90% of container capacity (“manager technique” described by Dumroese et al., 2015). Throughout the nursery production phase of the experiment, greenhouse conditions were maintained at 29.4/18.3°C (day/night), and natural light was supplemented as needed to maintain an 18 h photoperiod.

On 4 August 2015, the twelve racks (*i.e.*, experimental units) were randomly assigned to a block (n = 4) and irrigation treatment (described below), and irrigation treatments were initiated. Subirrigation was applied on a per rack basis. Irrigation treatments consisted of one of three irrigation levels (High = 90%, Medium = 80%, and Low = 70% of container capacity). These levels were chosen based upon a pilot study which found that the permanent wilting point for these seedlings occurred at ~65% container capacity (data not shown). New rack weights at container capacity were recorded for all racks. For the remainder of the irrigation portion of the experiment, racks were weighed daily for purposes of determining irrigation needs in accordance with irrigation treatments.

Despite different levels of irrigation, all seedlings received equal quantities of fertilizer over the course of the growing season, with each seedling receiving a total of 63 mg N during the irrigation treatment phase of this study. All fertilizer was applied as an aqueous solution *via* subirrigation. Of the total amount of fertilizer applied, the first two fertilizer applications each consisted of a solution of “starter fertilizer” (Peters Professional 10-30-20 Plant Starter, ICL Specialty Fertilizers, Dublin, OH, USA) and the remaining applications consisted of “grower fertilizer” (Peters Excel 21-5-20 Multi Purpose No Boron, ICL Specialty Fertilizers, Dublin, OH, USA).

### Synopsis of Morphological and Physiological Measurements

Irrigation treatments concluded on 6 October 2015, and all blocks were irrigated at this time. On 7–8 October, after 9 weeks of treatment implementation, a total of five seedlings were randomly sub-sampled from each experimental treatment unit for measurements and processing as described below. No trees were selected from the border rows of the racks. Morphological measurements were taken on all five sub-sampled seedlings, including seedling height, root collar diameter, total leaf area, stomatal density, total seedling dry mass, organ dry mass (roots, stems, and leaves), root:shoot (g:g), and average diameter of xylem vessels (physiologically active and total). Physiological measurements were also taken at this time and included net photosynthetic rates, transpiration rates, soluble sugar concentrations and contents by organ (roots, stems, and leaves), starch concentrations and contents by organ (roots, stems, and leaves), xylem flow velocity, the proportion of xylem which was physiologically active, and *δ* ^13^C of stem tissue as an integrated reflection of cumulative stomatal limitations on gas exchange. Measurements were conducted in the order presented below.

### Gas Exchange

Net photosynthesis and transpiration rates were measured on a south-facing, sun-exposed, mid-canopy leaf of five randomly sub-sampled seedlings per experimental treatment unit. Measurements occurred between 11:00 and 15:00 using an LI-6400XT Portable Photosynthesis System equipped with a 6400-02B LED light source (LI-COR, Inc., Lincoln, NE, USA). Instrument settings were as follows: block temperature = 20°C, CO_2_ reference = 375 µmol mol^−1^, flow rate = 500 µmol s^−1^, and photosynthetically active radiation in the leaf chamber (PAR) = 1,500 µmol m^−2^ s^−1^.

### General Morphological Characteristics

Total seedling height (measured from the root collar to the base of the pseudo-terminal bud) and root collar diameter were measured on each of the five sub-samples selected for the gas exchange measurements. Of the five sub-samples initially selected, three seedlings were randomly chosen for root morphology measurements. Seedlings were removed from their containers, roots were washed free from media, and root volume was measured gravimetrically using the water displacement method (Burdett, 1979).

### Xylem Characteristics and Stem Conductance

Of the three sub-samples used for root volume, two seedlings were randomly chosen for the assessment of xylem flow velocity, the proportion of xylem which was physiologically active, the average diameter of xylem vessels, both physiologically active and total (see Jacobsen et al., 2018). For these transpirational staining measurements, seedling stems were severed underwater at the root collar, the root systems were set aside for subsequent biomass determination (described below), and the intact aboveground portion of seedlings were suspended with the cut stem bases submerged 1 cm in 4.5 ml cuvettes containing 4.0 ml of a 0.01% (w:v) pH 2.0 crystal violet dye solution beneath an artificial light source providing 540 µmol m^−2^ s^−1^ PAR and an ambient temperature of 29.4°C. Seedlings were maintained in the dye solution for 60 s, after which they were transferred to a cuvette containing deionized water for 5 min maintained under identical light and temperature conditions. After removal from the deionized water cuvettes, a cross-section of stem approximately 0.5 mm in thickness was excised from each seedling at 2 cm above the root collar for microscopic analysis of xylem properties. Excised stem cross-sections were digitally photographed using a 4× objective lens under a light microscope (AmScope, MU1400). For each stem cross-section, the proportion of xylem which was physiologically active (as indicated by the presence of dye), the average diameter of physiologically active xylem vessels (those which were dyed), and the average diameter of all xylem vessels were determined from the digital images using Photoshop CC (Adobe Systems Inc., San Jose, CA, USA). Xylem flow velocity was determined by severing each stem at 1 cm increments using a razor blade, moving upward from the root collar, and visually identifying (with the aid of a 10× hand lens) the upper limit of dye occurrence within each seedling.

Of the two remaining sub-samples, one was randomly chosen to measure native stem conductance and leaf specific conductance. Stem segments 5 cm in length were severed at the root collar under water with a razor blade. Bark was removed and both ends were re-trimmed underwater with a fresh razor blade. Conductance was measured using the same technique as Kavanagh and Zaerr (1999), where stems were fitted to solution-filled tubing with a reservoir placed 0.5-0.6 m above the sample. The solution was filtered, deionized, and degassed. A pre-weighed, 4.5 ml cuvette with cotton gauze was used to capture solution passing through the stem. The vial was measured at 120 s intervals. Stem conductance (K_s_) was calculated by dividing the flow rate across the segment by the pressure gradient and stem cross sectional area. Leaf specific (K_l_) conductance was calculated similarly using the leaf area, measurements described below.

### Leaf Parameters

Leaves were subsequently removed from the two seedlings which had been sub-sampled for xylem measurements, as well as the third seedling sub-sampled for root volume. Leaves from each of these three sub-samples were placed on a flatbed scanner to determine total leaf area. Stomatal density was measured on the leaf from the gas exchange measurements. Images of stomata were taken digitally from a 10× objective lens under a light microscope (AmScope, MU1400) and analyzed using Photoshop CC.

### Biomass

The leaves, stems, and roots of the three sub-samples were placed in separate paper bags and oven-dried at 68°C for 48 h. Dry masses were recorded for each organ of each sub-sample, and tissues were ground in a Wiley mill (Thomas Scientific, Swedesboro, NJ, USA) to pass a 20-mesh screen. Total seedling mass was calculated as the sum of the individual organ mass for each sub-sampled seedling, and root:shoot was calculated by dividing the root mass by the sum of the stem and leaf masses for that seedling. Specific leaf area was calculated as total leaf area divided by total leaf mass per seedling.

### Non-Structural Carbohydrates

Soluble sugar concentrations were determined for each organ of each of the three sub-samples. In short, ethanol-soluble sugars were extracted from a 50 mg aliquot of dried and ground tissue from each organ of each sub-sampled seedling *via* three sequential extractions using 1 ml of room-temperature 80% (v:v) ethanol for each extraction. Supernatant from each of the three extractions was pooled, and an aliquot of the pooled supernatant was diluted and subjected to an anthrone assay for colorimetric quantification of soluble sugar concentration (Sloan and Jacobs, 2012). Soluble sugar contents of each organ of each sub-sampled seedling were calculated by multiplying the soluble sugar concentration of an organ by its mass. After soluble sugars were extracted, the residual tissue was again oven-dried at 68°C for 48 h prior to the analysis of starch concentration.

Starch concentrations were determined for each of the three sub-sampled seedlings. Starch contained within the oven-dried residual tissue from which soluble sugars had been extracted was enzymatically digested into glucose with α-amylase and amyloglucosidase prior to extraction and colorimetric quantification *via* a modified Trinder assay (Trinder, 1969; Lott and Tuner, 1975; Schwab and Lindell, 2010). Starch contents of each organ of each sub-sampled seedling were calculated by multiplying the starch concentration of an organ by its mass.

### Carbon Isotope Ratio (*δ* ^13^C)

Aliquots of ground stem tissue from the three sub-samples were submitted to the Stable Isotope Core Laboratory of Washington State University for determination of *δ* ^13^C. Samples for carbon were converted to CO_2_ with an elemental analyzer (ECS 4010, Costech Analytical, Valencia, CA) and then separated with a 3 m GC column and analyzed with a continuous flow isotope ratio mass spectrometer (Delta PlusXP, Thermofinnigan, Bremen). Isotope reference materials were interspersed with samples for calibration. Carbon isotope ratios were expressed as *δ* ^13^C relative to the Pee Dee River belemnite standard (Craig, 1957).

### Statistical Design and Analyses

This experiment was designed and analyzed as a 3 × 3 factorial randomized complete block design, with three seed source levels and three irrigation levels. Within each of the four blocks (*i.e.*, four replicates), each treatment combination was represented by a single complete rack of seedlings which had been randomly assigned to a block and irrigation level. All data were analyzed using the PROC MIXED procedure in SAS (SAS Institute Inc., Cary, NC, USA) with *α* = 0.05. Tukey’s Honest Significant Difference test was used to detect significant differences between means (*α* = 0.05). When interactions were found to be non-significant, lower order terms were reported. All residuals were checked for constant variance and normality. Both K_s_ and K_l_ data were log transformed for analysis to meet the assumptions of constant variance and normality. Data are back transformed for manuscript presentation.

## Results

### General Morphological Characteristics and Biomass

No interactions between seed source and irrigation treatment were observed for seedling total mass, leaf mass, stem mass, root mass, total seedling height, root collar diameter, or root-to-shoot ratio by mass (p > 0.05). Seed source was not found to influence leaf, stem, or total seedling mass (p = 0.9670, 0.8688, and 0.0573, respectively); however, it significantly influenced root mass, total height, root collar diameter, and root:shoot (p = 0.0119). Root mass differed between all seed sources, with the AB source exhibiting the highest and the NM source exhibiting the lowest root mass (**Table 1**). Root:shoot exhibited the same pattern across sources as root mass, while total height was found to have an inverse pattern by source (**Table 1**). Root collar diameter was largest in the UT source and lowest in the AB source, with no difference observed between the NM seed source and other sources (**Table 1**).

**Table 1:** Aspen seedling morphological characteristics by seed source **(A)** and irrigation treatment **(B)**.

A. Morphological responses by seed source			
Morphological parameter	Seed source		
	AB	UT	NM
Total seedling mass (g)	2.02 (0.11)a	1.69 (0.15)a	1.55 (0.16)a
Leaf mass (g)	0.60 (0.04)a	0.59 (0.06)a	0.59 (0.05)a
Stem mass (g)	0.52 (0.04)a	0.54 (0.06)a	0.56 (0.06)a
Root mass (g)	0.90 (0.06)a	0.56 (0.06)b	0.39 (0.05)c
Total height (cm)	23.0 (1.63)c	29.1 (1.02)b	37.6 (1.98)a
Root collar diameter (mm)	2.9 (0.14)b	3.3 (0.11)a	3.2 (0.14) ab
Root : Shoot (g:g)	0.83 (0.06)a	0.48 (0.01)b	0.32 (0.02)c
**B. Morphological responses by irrigation treatment**			
**Morphological parameter**	**Irrigation treatment**		
****	**High**	**Medium**	**Low**
Total seedling mass (g)	1.64 (0.17)a	1.79 (0.17)a	1.82 (0.10)a
Leaf mass (g)	0.51 (0.04)a	0.63 (0.06)a	0.65 (0.04)a
Stem mass (g)	0.50 (0.05)a	0.53 (0.06)a	0.60 (0.05)a
Root mass (g)	0.63 (0.10)a	0.64 (0.09)a	0.58 (0.06)a
Total height (cm)	28.8 (2.01)b	27.2 (2.37)b	33.8 (2.36)a
Root collar diameter (mm)	3.0 (0.15)a	3.1 (0.15)a	3.3 (0.11)a
Root : Shoot (g:g)	0.59 (0.08)a	0.55 (0.07)a	0.49 (0.06)a

Values displayed are the mean (± standard error of the mean) of total seedling mass, leaf mass, stem mass, root mass, height, root collar diameter, and root:shoot. Seed source: Alberta (AB), Utah (UT), and New Mexico (NM). Irrigation abbreviations: High (90% saturation), Medium (80% saturation), and Low (70% saturation). Within each parameter, means followed by the same letter do not differ significantly (α = 0.05).

Irrigation treatments did not influence total seedling mass, leaf mass, stem mass, root mass, root collar diameter, or root-to-shoot ratio (p = 0.6102, 0.0774, 0.4282, 0.7234, 0.1433, and 0.1625, respectively). However, total height responded positively to decreasing water availability, with the Low treatment exhibiting the greatest total height (p = 0.0009; **Table 1**).

### Leaf Parameters and Gas Exchange

No interactions (p > 0.05) were observed for net photosynthetic rates, specific leaf area (SLA), stomatal density (adaxial and abaxial). Seed source did not influence net photosynthetic rates (p = 0.1836), although it significantly influenced SLA (p = 0.0253) and stomatal density (abaxial: p = 0.0223; adaxial: p = 0.007). SLA was higher in the NM source than in the AB source, while the UT source did not differ from either the NM or AB source. Abaxial stomatal density was higher in the UT source than in the AB source, while adaxial stomatal density was higher in the UT source than in the NM source. No differences in stomatal density were observed between the AB and NM sources (**Table 2**).

**Table 2:** Aspen leaf parameter responses by seed source **(A)** and irrigation treatment **(B)**.

A. Leaf parameter responses by seed source			
Leaf parameter	Seed source		
	AB	UT	NM
A_net_ (µmol m^−2^ s^−1^)	10.93 (0.97)a	10.00 (0.71)a	11.67 (0.86)a
SLA (mm^2^ g^−1^)	273.9 (7.5)b	309.4 (15.8)ab	324.2 (16.4)a
Abaxial stomatal density	156.4 (14.3)b	222.7 (17.0)a	214.4 (19.5)ab
Adaxial stomatal density	38.7 (4.6)ab	50.3 (4.9)a	27.1 (6.8)b
**B. Leaf parameter responses by irrigation treatment**			
**Leaf parameter**	**Irrigation treatment**		
	**High**	**Medium**	**Low**
A_net_ (µmol m^−2^ s^−1^)	9.21 (0.69)a	9.91 (0.53)a	13.48 (0.78)b
SLA (mm^2^ g^−1^)	322.6 (17.6)a	300.2 (16.5)a	284.8 (6.9)a
Abaxial stomatal density	210.6 (16.7)a	196.1 (20.1)a	186.1 (19.9)a
Adaxial stomatal density	43.1 (5.4)a	32.2 (6.8)a	40.9 (5.9)a

Values displayed are the mean (± standard error of the mean) of net photosynthetic rate (A_net_), specific leaf area (SLA), stomatal density of the lower side of the leaf (abaxial), and stomatal density of the upper side of the leaf (adaxial). Seed source abbreviations: Alberta (AB), Utah (UT), and New Mexico (NM). Irrigation abbreviations: High (90% saturation), Medium (80% saturation), and Low (70% saturation). Within each parameter, means followed by the same letter do not differ significantly (α = 0.05).

Irrigation treatments were not found to influence SLA, abaxial stomatal density, or adaxial stomatal density (p = 0.1227, 0.6079, and 0.2441, respectively). Irrigation treatments, however, significantly increased net photosynthetic rates in the Low treatment relative to the Medium and High treatments (p < 0.0001; **Table 2**).

### Xylem Characteristics and Stem Conductance

**Figure 1:**
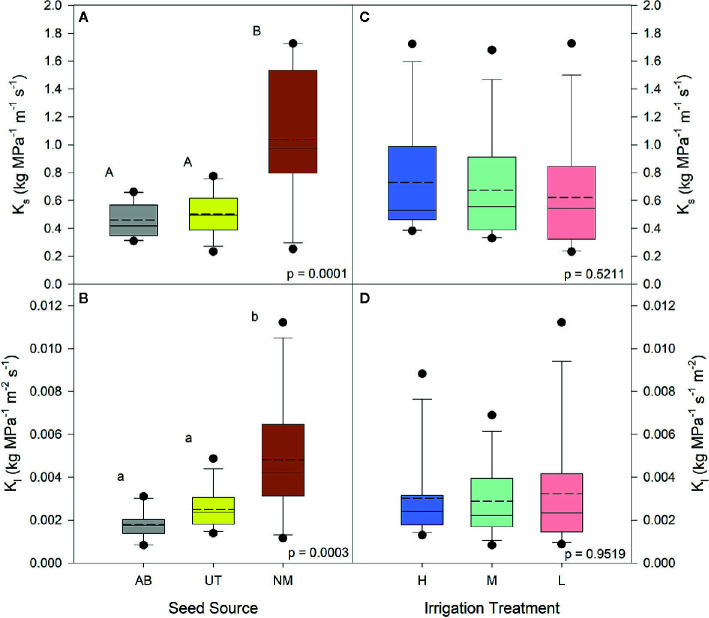
Aspen seedling stem conductance by seed source and irrigation treatment. Native stem conductance (Ks) is shown in **(A**, **C)**; leaf specific stem conductance is shown in **(B** ****, **D)**. Dashed lines are mean stem conductance K_1_; solid lines are the median. Seed source: Alberta (AB), Utah (UT), and New Mexico (NM). Irrigation: High (90% saturation), Medium (80% saturation), and Low (70% saturation). Within each parameter, means followed by the same letter do not differ significantly (*α* = 0.05).

No interactions between seed source and irrigation treatment were observed for the percentage of physiologically active xylem, average xylem vessel diameter, average diameter of physiologically active xylem vessels, or xylem flow velocity (p > 0.05). Both K_s_ and K_l_ data yielded no interactions (p < 0.5440). Seed source was not found to influence the average diameter of physiologically active xylem vessels (p = 0.4070) or xylem flow velocity (p = 0.3128), but the NM seed source was found to have a higher percentage of physiologically active xylem compared to the UT source and a larger average xylem vessel diameter than other seed sources (p = 0.0276 and p = 0.0008, respectively; **Table 3**). Seed source elicited a significant response for both K_s_ and K_l_ (p < 0.0003). For each, the AB and UT were similar in value and exhibited less conductance than the NM source (**Figures 1A, B**).

**Table 3:** Aspen seedling xylem characteristics by seed source **(A)** and irrigation treatment **(B)**.

A. Xylem responses by seed source			
Xylem parameter	Seed source		
	AB	UT	NM
% active xylem	11.5 (1.9)ab	11.1 (3.0)b	19.2 (2.6)a
Average xylem diameter (µm)	25.8 (0.8)b	26.6 (0.8)b	29.6 (0.7)a
Average active diameter (µm)	28.3 (1.0)a	27.2 (1.6)a	29.9 (1.7)a
Xylem flow velocity (cm h^−1^)	54.6 (3.9)a	45.0 (7.1)a	47.1 (3.9)a
**B. Xylem responses by irrigation treatment**			
**Xylem parameter**	**Irrigation treatment**		
	**High**	**Medium**	**Low**
% active xylem	9.9 (1.8)b	12.9 (2.8)ab	19.2 (2.8)a
Average xylem diameter (µm)	26.0 (0.9)a	27.9 (0.8)a	28.1 (0.9)a
Average active diameter (µm)	26.6(1.9)a	29.1 (1.4)a	29.8 (0.9)a
Xylem flow velocity (cm h^−1^)	40.4 (2.9)b	45.4 (3.9)ab	60.6 (6.4)a

Values displayed are the mean (± standard error of the mean) of the percentage of xylem which was physiologically active (% active xylem), the average diameter of xylem vessel elements (average xylem diameter), the average diameter of physiologically active xylem vessel elements (average active diameter), and xylem flow velocity. Seed source abbreviations: Alberta (AB), Utah (UT), and New Mexico (NM). Irrigation abbreviations: High (90% saturation), Medium (80% saturation), and Low (70% saturation). Within each parameter, means followed by the same letter do not differ significantly (α = 0.05).

**Figure 2:**
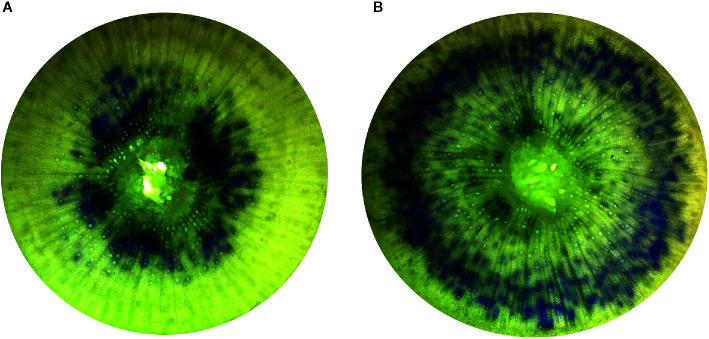
Comparison of physiologically active xylem of first year *Populus tremuloides* seedlings by irrigation treatment. The blue stained (crystal violet dye) areas indicate those xylem vessels that are actively conductive. The images displayed are representative of the High irrigation treatment level **(A)** and Low irrigation treatment level **(B)**.

Irrigation treatments were not found to influence the average diameter of physiologically active xylem vessels (p = 0.2686) or the average xylem vessel diameter (p = 0.0622). The percentage of physiologically active xylem (p = 0.0219) and xylem flow velocity (p = 0.0104) were found to increase with decreasing irrigation in the nursery (**Figure 2**). The Low irrigation level exhibited the highest values for each of these parameters (**Table 3**). Irrigation treatment had no effects on either K_s_ or K_l_ (p > 0.5211; **Figures 1C, D**).

### Non-Structural Carbohydrates

No interactions between seed source and irrigation treatments were observed for any measured non-structural carbohydrate parameter (p > 0.05). Seed source was not found to influence leaf starch concentration, leaf sugar content, stem sugar content, or leaf starch content (p = 0.0511, 0.4774, 0.1713, and 0.1765; respectively). However, seed source significantly influenced leaf sugar concentration, stem sugar concentration, root sugar concentration, stem starch concentration, root starch concentration, root sugar content, stem starch content, and root starch content (p ≤ 0.0037). Seed sources exhibited trends of decreasing non-structural carbohydrate concentrations and contents with decreasing latitudes of origin (**Tables 4** and **5**).

**Table 4:** Aspen seedling non-structural carbohydrate concentrations by seed source and irrigation treatment.

A. Soluble sugar concentrations by seed source (mg g^−1^)			
Organ	Seed source		
	AB	UT	NM
Leaves	128.2 (3.7)a	122.6 (4.5)a	110.7 (3.2)b
Stems	85.4 (2.7)a	70.5 (3.8)b	57.9 (3.5)c
Roots	56.8 (1.7)a	44.7 (2.9)b	33.7 (1.8)c
**B. Starch concentrations by seed source (mg g^−1^)**			
**Organ**	**Seed source**		
****	**AB**	**UT**	**NM**
Leaves	46.5 (6.0)a	55.9 (10.5)a	32.0 (5.5)a
Stems	65.6 (5.4)a	44.0 (4.4)b	23.8 (4.9)c
Roots	195.0 (7.7)a	101.4 (7.2)b	63.8 (7.1)c
**C. Soluble sugar concentrations by irrigation treatment (mg g^−1^)**			
**Organ**	**Irrigation treatment**		
****	**High**	**Medium**	**Low**
Leaves	120.2 (4.1)a	120.0 (4.0)a	121.4 (5.0)a
Stems	75.6 (4.2)a	72.4 (5.6)ab	65.8 (3.9)b
Roots	45.7 (3.2)a	45.7 (4.0)a	43.8 (3.5)a
**D. Starch concentrations by irrigation treatment (mg g^−1^)**			
**Organ**	**Irrigation treatment**		
****	**High**	**Medium**	**Low**
Leaves	49.0 (7.6)a	49.9 (9.0)a	35.5 (7.4)a
Stems	57.0 (6.4)a	38.3 (6.8)b	38.0 (6.6)b
Roots	128.2 (17.3)a	112.1 (16.3)a	120.0 (20.4)a

Values displayed are the mean (± standard error of the mean) of the soluble sugar concentrations by organ and seed source **(A)**, starch concentrations by organ and seed source **(B)**, soluble sugar concentrations by organ and irrigation treatment **(C)**, and starch concentrations by organ and irrigation treatment **(D)**. Seed source abbreviations: Alberta (AB), Utah (UT), and New Mexico (NM). Irrigation abbreviations: High (90% saturation), Medium (80% saturation), and Low (70% saturation). Within each parameter, means followed by the same letter do not differ significantly (α = 0.05).

**Table 5:** Aspen seedling non-structural carbohydrate contents by seed source and irrigation treatment.

A. Soluble sugar contents by seed source (mg)			
Organ	Seed source		
	AB	UT	NM
Leaves	78.6 (5.6)a	73.4 (7.6)a	68.1 (7.2)a
Stems	44.6 (2.8)a	41.7 (5.3)a	33.9 (3.8)a
Roots	52.0 (3.6)a	28.1 (3.8)b	14.7 (2.0)c
**B. Starch contents by seed source (mg)**			
**Organ**	**Seed source**		
****	**AB**	**UT**	**NM**
Leaves	32.1 (4.4)a	40.2 (9.9)a	21.9 (5.7)a
Stems	33.2 (2.7)a	25.3 (3.9)a	12.6 (2.0)b
Roots	173.8 (13.6)a	61.9 (7.3)b	29.1 (5.2)c
**C. Soluble sugar contents by irrigation treatment (mg)**			
**Organ**	**Irrigation treatment**		
****	**High**	**Medium**	**Low**
Leaves	62.5 (5.7)a	77.2 (7.7)a	80.4 (6.1)a
Stems	33.9 (3.9)a	40.4 (5.1)a	41.0 (3.8)a
Roots	32.8 (6.6)a	33.2 (5.8)a	28.8 (4.3)a
**D. Starch contents by irrigation treatment (mg)**			
**Organ**	**Irrigation treatment**		
****	**High**	**Medium**	**Low**
Leaves	28.9 (5.5)a	38.8 (9.1)a	26.5 (6.6)a
Stems	28.9 (3.9)a	20.7 (3.7)a	21.4 (3.7)a
Roots	97.4 (24.0)a	85.9 (18.8)a	81.5 (19.2)a

Values displayed are the mean (± standard error of the mean) of the soluble sugar contents by organ and seed source **(A)**, starch contents by organ and seed source **(B)**, soluble sugar contents by organ and irrigation treatment **(C)**, and starch contents by organ and irrigation treatment **(D)**. Seed source abbreviations: Alberta (AB), Utah (UT), and New Mexico (NM). Irrigation abbreviations: High (90% saturation), Medium (80% saturation), and Low (70% saturation). Within each parameter, means followed by the same letter do not differ significantly (α = 0.05).

Irrigation treatments did not influence leaf or root sugar concentrations (p = 0.9527 and 0.7851, respectively) or leaf or root starch concentrations (p = 0.2453 and 0.2766, respectively). Additionally, irrigation did not influence leaf, stem, or root sugar contents (p = 0.1001, 0.9275, and 0.5755, respectively) or leaf, stem, or root starch contents (p = 0.4058, 0.0676, and 0.4651, respectively). Only stem sugar and starch concentrations (p = 0.0293 and p = 0.0009, respectively) were influenced by irrigation treatments, with stem sugar concentrations decreasing in the Low treatment relative to the High and stem starch concentrations decreasing in both the Medium and Low treatments relative to the High treatment (**Tables 4** and **5**).

### Carbon Isotope Ratio (*δ* ^13^C)

**Figure 3:**
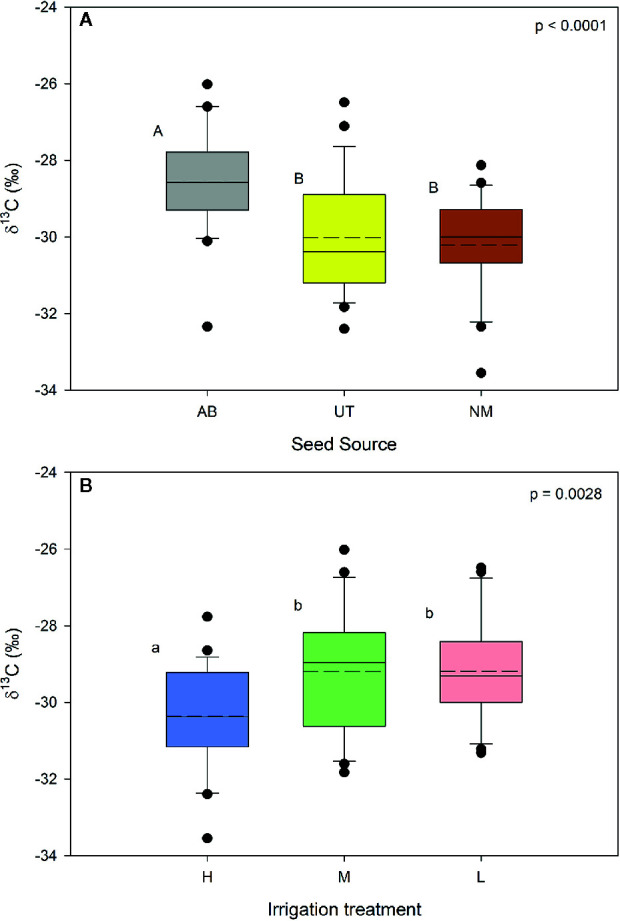
Aspen seedling stem tissue *δ* ^13^C by seed source **(A)** and irrigation treatment **(B)**. Dashed lines are mean seedling stem tissue *δ* ^13^C; solid lines are the median. Seed source: Alberta (AB), Utah (UT), and New Mexico (NM). Irrigation: High (90% saturation), Medium (80% saturation), and Low (70% saturation). Within each parameter, means followed by the same letter do not differ significantly (*α* = 0.05).

No significant interaction occurred between seed source and irrigation treatments for *δ* ^13^C of stem tissues (p = 0.5933). Seed source and irrigation main effects were significant (p < 0.0001 and p = 0.0028, respectively). Within seed source, the AB source exhibited a less negative *δ* ^13^C compared to the other two sources (**Figure 3A**). No differences were found between NM and UT sources. For the irrigation treatments, *δ* ^13^C in the High irrigation level was found to be significantly more negative compared to the Medium and Low levels (**Figure 3B**).

## Discussion

### Impacts of Irrigation Limitations

Studies that have limited irrigation during nursery production to produce drought-conditioned seedlings have been highly varied in their implementation and results. The initiation of drought conditioning treatments has begun at a range of phenological stages of seedling development and has been implemented across different lengths of time, likely contributing to the varied results (Zwiazek and Blake, 1989; van den Driessche, 1991; Villar-Salvador et al., 1999; Vilagrosa et al., 2003; Galvez et al., 2011). It is known that xylem begins to develop and differentiate early in a seedling’s development (0–10 weeks after germination; Miller and Johnson, 2017), but the earliest initiation of a drought-conditioning treatment identified by the authors was reported by Galvez et al. (2011) at 14 weeks after germination. Therefore, our study is novel in that its drought conditioning treatments began early in the period of xylem development (approximately 4 weeks after germination) and continued throughout the full nursery growing season.

In the present study, patterns of height growth and net photosynthesis differed substantially from those described by Galvez et al. (2011), which found that seedlings in the well-watered control treatment were much taller and had greater net photosynthetic rates than seedlings in the drought treatment. Typical seedling photosynthetic rates follow a pattern where they are low in times of limited moisture availability and exhibit the opposite when water is more available (Pinto et al., 2012). We suspect, however, that irrigation timing prior to sampling may have impacted the rates seen in this study. Pinto et al. (2016) observed that moisture-limited seedlings exhibited low photosynthetic rates, but that these rates quickly rebounded with an increase in soil moisture. The same may have been true for the seedlings in this study that were just irrigated (i.e., rehydrated) prior to measuring photosynthesis. These seedlings were subjected to dehydration/rehydration cycles as a result of the irrigation regimes throughout the growth cycle. Increased photosynthetic rates of the low irrigation treatment may have been an opportunistic response to the rehydration portion of the irrigation cycle, thus contributing to the overall lack of growth differences seen in our results. This is further evidenced by the trend in *δ* ^13^C values, which is discussed below. It is also suspected that the significantly reduced height growth and photosynthetic rates described by Galvez et al. (2011) reflect the typical responses of seedlings when they are exposed to drought after being produced in a nursery under non-limiting irrigation regimes. In contrast, the increased height growth and photosynthetic rates of seedlings in the Low irrigation treatment reflect the responses of seedlings whose physiology and anatomy have been altered by exposure to cyclic moisture limitations beginning at an early developmental stage, potentially leading to an improved ability to respond opportunistically to ephemeral increases in soil moisture.

Soluble sugar concentrations of stems were lower in seedlings from the Low irrigation treatment compared with seedlings from the High irrigation treatment, while starch concentrations were lower in seedlings of both the Low and Medium irrigation treatments relative to seedlings of the High irrigation treatment (**Table 4**). Considering that no differences were observed between irrigation levels for stem sugar or starch contents, it is likely that the reductions observed in the Low treatment were a growth dilution effect resulting from the increased seedling heights observed in this treatment (**Tables 1** and **4**). In examining the response of soluble sugar and starch concentrations of one-year-old aspen seedlings to drought, Galvez et al. (2011) found a pattern of increasing root starch concentrations in seedlings exposed to drought, which seemed to correspond to the observed cessation of aboveground growth in these seedlings. In contrast, the present study found no difference in non-structural carbohydrate concentrations or contents in roots of drought-conditioned seedlings, which seemed to correspond to the absence of negative impacts on above-ground growth in drought-conditioned seedlings in our study. This again highlights the different physiological responses to drought observed in seedlings which have been grown under drought conditions from an early age as opposed to those grown under non-limiting soil moisture and only later exposed to drought conditions.

Unfortunately, neither soil nor plant water potentials were directly monitored during this study. As a proxy, stable carbon isotopes were used as a time-integrated index of the ratio of intercellular to ambient CO_2_ concentration to infer water availability and water-use efficiency (Ehleringer and Osmond, 1989; Warren et al., 2001; Dawson et al., 2002). The observation that *δ* ^13^C values of stem tissues were enriched in the Medium and Low irrigation treatments relative to the High irrigation treatments (**Figure 3B**) confirms that both the Medium and Low irrigation treatments induced levels of water limitation sufficient to cause stomatal closure and isotopic enrichment during periods of water-limited photosynthesis. Stable carbon isotopes have also been used to confirm water relations and water stress for nursery produced seedlings by Pinto et al. (2012).

Though irrigation had a notable effect on *δ* ^13^C values, morphological parameters were minimally affected. It is suspected that though stomata may have been closed more for the Medium and Low irrigation treatments, the difference was made up with increased photosynthesis. Studies have shown that leaf photosynthetic traits can scale with hydraulic conductivity in large trees (Bodribb and Field, 2000; Santiago et al., 2004), though, this study did not show increased K_s_ and K_l_ for the Medium and Low treatments. Interestingly, higher photosynthesis in the Low treatment did correspond to increased active xylem percentage and xylem flow velocity. Xylem vessel development in nursery *versus* natural settings is minimally explored; nevertheless, it is known that the functional attributes of xylem vessels can change over the lifespan of the plant (Jacobsen et al., 2018). The attributes measured in this study represent merely a snapshot in developmental time. Despite this, the data may suggest that the observed patterns of adaptations to decreased water availability in quaking aspen seedlings rely heavily on the modification of the physiology of above-ground portions of the plant in an effort to facilitate water movement and prevent xylem cavitation events, with negligible modification of the below-ground portions of the plant or overall biomass allocation. It is suspected that the increased active xylem may represent a buffer to cavitation events for seedlings grown in water limiting environments (Jacobsen et al., 2015). Seed source data may corroborate this. NM sources illustrated higher K_S_, K_l_, percent active xylem, and average xylem diameter traits. Of the three sources, NM receives the least amount of annual precipitation, and most of this comes with the summer monsoons when the species has leaves and is actively growing. It is logical that hydraulic systems for this region are built with a pulse-activated and highly buffered system for physiological functioning.

Understanding such aspects of the strategies by which aspen seedlings attempt to adapt to environmental conditions is important for nursery managers and restoration practitioners inasmuch as it can help inform them as to the likely influence of nursery cultural practices and post-transplant environmental characteristics on seedling development and survival.

### Relevance for Nursery Management and Outplanting

The general lack of interactions suggests that the observed responses to the irrigation treatments may remain consistent across a range of seed sources for aspen. As a result, there is the potential for nursery managers to use these irrigation strategies in their nursery programs with similar outcomes for a range of seed sources for aspen. Although further investigations will be necessary to confirm the uniformity of the effects of irrigation limitations during nursery culture across a much broader range of seed sources, as well as to confirm the effects of such irrigation limitations for drought-conditioning of other species, the absence of interactions lends to the idea these traits exhibit some plasticity across seed sources. This leaves open the possibility that such irrigation treatments may be used to produce drought-conditioned nursery stocktypes, thereby providing tree seedling nursery managers with a novel cultural approach that allows for the production of new seedling stocktypes culturally pre-conditioned to a variety of outplanting site moisture regimes. This expansion and adaptation of nursery cultural practices would be a logical application of the Target Plant Concept and would constitute a step forward for nurseries seeking to develop more advanced stocktypes that are potentially better adapted to harsh outplanting sites (Landis, 2011; Dumroese et al., 2016). Such drought-conditioned stocktypes, in turn, could have the potential to substantially improve the success of reforestation and afforestation operations on harsh, moisture-limited outplanting sites.

## Conclusions

Limitation of irrigation during nursery production was found to influence a small number of above-ground traits in aspen seedlings, resulting in increased height growth, elevated photosynthetic rates, increased percentages of active xylem, and increased xylem flow velocities. No influence on measured below-ground plant properties was observed, and few interactions were observed between seed source and irrigation treatments. This suggests that 1) adaptation to drought in aspen seedlings may rely primarily on alteration of above-ground plant traits to improve water use efficiency, and 2) that observations from this study may be applicable across a range of seed sources. Follow-up studies are needed to determine the extent to which the altered seedling characteristics observed in the Low treatment of this study translate into altered patterns of seedling survival, growth, and physiology following outplanting into water-limited environments. This research suggests the possibility of developing drought-conditioned nursery stocktypes that are pre-conditioned to harsh, drought-prone planting sites.

## Data Availability Statement

The raw data supporting the conclusions of this article will be made available by the authors, without undue reservation.

## Author Contributions

OB, JS, and JP all contributed equally to the design, implementation, analysis, and writing of this research.

## Funding

This work was supported in part by the USDA National Institute of Food and Agriculture, McIntire Stennis project 1002447.

## Conflict of Interest

The authors declare that the research was conducted in the absence of any commercial or financial relationships that could be construed as a potential conflict of interest.
