# Supplementary material for: Drought-Conditioning of Quaking Aspen (Populus tremuloides Michx.) Seedlings During Nursery Production Modifies Seedling Anatomy and Physiology
Source: Front Plant Sci. 2020 Sep 4;11:557894. doi: 10.3389/fpls.2020.557894 (PMC7498648; doi:10.3389/fpls.2020.557894)
Supplement: Supplementary file 1 [file Table_1.docx]

Supplementary Tables:

| Table S1. |  |  |  |
| --- | --- | --- | --- |
| Source of variation for morphological responses |  |  |  |
| Morphological parameter | Seed source | Irrigation | Interaction |
| Total seedling mass (g) | 0.0573 | 0.6102 | p > 0.05 |
| Leaf mass (g) | 0.9670 | 0.0774 | p > 0.05 |
| Stem mass (g) | 0.8688 | 0.4282 | p > 0.05 |
| Root mass (g) | < 0.0001 | 0.7234 | p > 0.05 |
| total height (cm) | < 0.0001 | 0.0009 | p > 0.05 |
| Root collar diameter (mm) | 0.0119 | 0.1433 | p > 0.05 |
| Root:Shoot (g:g) | < 0.0001 | 0.1625 | p > 0.05 |
|  |  |  |  |

| Table S2. |  |  |  |
| --- | --- | --- | --- |
| Source of variation for leaf parameter responses |  |  |  |
| Leaf parameter | Seed source | Irrigation | Interaction |
| Anet (µmol m-2 s-1) | 0.1836 | < 0.0001 | p > 0.05 |
| SLA (mm2 g-1) | 0.0253 | 0.1227 | p > 0.05 |
| Abaxial stomatal density | 0.0223 | 0.6079 | p > 0.05 |
| Adaxial stomatal density | 0.0070 | 0.2441 | p > 0.05 |

| Table S3. |  | |  | |  | |
| --- | --- | --- | --- | --- | --- | --- |
| Source of variation for xylem responses by seed source | |  | |  | |  |
| Xylem parameter | Seed source | | Irrigation | | Interaction | |
| % active xylem | 0.0276 | | 0.0219 | | p > 0.05 | |
| Average xylem diameter (µm) | 0.0008 | | 0.0622 | | p > 0.05 | |
| Average active diameter (µm) | 0.4070 | | 0.2686 | | p > 0.05 | |
| Xylem flow velocity (cm h-1) | 0.3128 | | 0.0104 | | p > 0.05 | |

| Table S4. |  |  |  |
| --- | --- | --- | --- |
| Source of variation for  non-structural carbohydrate concentrations  A. Soluble sugar concentrations (mg g-1) | | |  |
| Organ | Seed source | Irrigation | Interaction |
| Leaves | 0.0037 | 0.9527 | p > 0.05 |
| Stems | < 0.0001 | 0.0293 | p > 0.05 |
| Roots | < 0.0001 | 0.7851 | p > 0.05 |
| B. Starch concentrations (mg g-1) | |  |  |
| Organ | Seed source | Irrigation | Interaction |
| Leaves | 0.0511 | 0.2453 | p > 0.05 |
| Stems | < 0.0001 | 0.0009 | p > 0.05 |
| Roots | < 0.0001 | 0.2766 | p > 0.05 |

| Table S5.  Source of variation for  non-structural carbohydrate contents | |  |  |
| --- | --- | --- | --- |
| A. Soluble sugar contents (mg) | |  |  |
| Organ | Seed source | Irrigation | Interaction |
| Leaves | 0.4774 | 0.1001 | p > 0.05 |
| Stems | 0.1713 | 0.9275 | p > 0.05 |
| Roots | < 0.0001 | 0.5755 | p > 0.05 |
| B. Starch contents (mg) |  |  |  |
| Organ | Seed source | Irrigation | Interaction |
| Leaves | 0.1765 | 0.4058 | p > 0.05 |
| Stems | < 0.0001 | 0.0676 | p > 0.05 |
| Roots | < 0.0001 | 0.4651 | p > 0.05 |
